# Cation Type Specific Cell Remodeling Regulates Attachment Strength

**DOI:** 10.1371/journal.pone.0102424

**Published:** 2014-07-11

**Authors:** Alexander Fuhrmann, Julie Li, Shu Chien, Adam J. Engler

**Affiliations:** 1 Department of Bioengineering, Institute of Engineering in Medicine, University of California San Diego, La Jolla, California, United States of America; 2 Department of Medicine, Institute of Engineering in Medicine, University of California San Diego, La Jolla, California, United States of America; 3 Sanford Consortium for Regenerative Medicine, La Jolla, California, United States of America; University of California, Berkeley, United States of America

## Abstract

Single-molecule experiments indicate that integrin affinity is cation-type-dependent, but in spread cells integrins are engaged in complex focal adhesions (FAs), which can also regulate affinity. To better understand cation-type-dependent adhesion in fully spread cells, we investigated attachment strength by application of external shear. While cell attachment strength is indeed modulated by cations, the regulation of integrin-mediated adhesion is also exceedingly complex, cell specific, and niche dependent. In the presence of magnesium only, fibroblasts and fibrosarcoma cells remodel their cytoskeleton to align in the direction of applied shear in an α_5_-integrin/fibronectin-dependent manner, which allows them to withstand higher shear. In the presence of calcium or on collagen in modest shear, fibroblasts undergo piecewise detachment but fibrosarcoma cells exhibit increased attachment strength. These data augment the current understanding of force-mediated detachment by suggesting a dynamic interplay *in situ* between cell adhesion and integrins depending on local niche cation conditions.

## Introduction

Integrin-mediated adhesion to extracellular matrix (ECM) occurs via complex molecular clusters called focal adhesions (FAs) that enable cells to transduce forces and signals to and from the cell's surroundings. Proteins within FAs are intrinsically dynamic, with average integrin bond lifetimes on the order of seconds; thus cell adhesion can only be achieved by the continuous binding, disengaging, and rebinding of many integrins to and from ECM, i.e. avidity. Single-molecule studies indicated that integrin binding affinity for ECM is highly influenced by niche conditions [1], especially the fluctuating concentrations and types of cations, specifically magnesium and/or calcium [2]; this effect can be as strong as those observed when inhibiting the activity of focal adhesion proteins [3]. Given the broad scope of cation-mediated cell processes [4], such reductionist experiments might be preferable; however, integrin affinity and avidity are internally regulated within FAs [5], and thus their response to cations may be different when studying integrins on a single-molecule level versus *in situ*. Events as basic as cell adhesion have long been known to be seriously compromised in the absence of cations, which facilitate ECM-ligand binding [6]–[9], and this behavior also may not be represented with single-molecule assays.

To address this knowledge gap, a variety of methods have been developed to quantify cell adhesion *in situ* after cell attachment. These range from bead binding assays (e.g. biomembrane force probes and optical tweezers) to whole cell-ECM interactions (e.g. micropipette aspiration and centrifugal or shear force assays); most methods apply force to dissociate bonds shortly after initial attachment to the substrate (from a few seconds to several minutes) [10], [11]. In contrast, fully adhered cells undergo adhesion strengthening by a complex interplay of integrin binding, focal adhesion assembly, and cell spreading over hours to days in culture [12], [13]. While theoretical models predict [12] and experimental data suggest [14] that fully adhered cells detach via peeling, reports commonly describe detachment in terms of a cell being either present on the substrate or not [15], [16]; it should be noted that peeling is different from active remodeling, which is observed over several hours of shear exposure [17] instead of minutes. Without experimental data confirming this model, the effects of peeling on the cell's ability to withstand shear remain unknown.

Although these cell-based assays allow cell-adhesion quantification in different cation environments, most studies appear to examine adhesion in the presence of high cation concentration, i.e. a phosphate buffered saline without defining cation composition [13], [18], which we assume contains high Mg^2+^ and Ca^2+^ concentrations consistent with prior work [15], [16]; while that ensures the maximal activation of integrins, it may not represent the most physiologically appropriate environment [19]. Within blood and most interstitial fluid, free cation levels are fairly homogeneously distributed at ∼0.6 mM Mg^2+^ and 1 mM Ca^2+^ [20]. In tissues, most of these cations are bound [21], and thus differences in free cation concentration can be easily altered during disease. For example after a stroke, serum concentrations as low as 0.3 mM Mg^2+^ have been reported [20]. Free calcium is also reduced immediately after spinal cord injury [22]. Conversely cations are more concentrated in human breast tumors than in adjacent stroma [23] but remain lower than in serum.

To understand the modulation of integrin function by a variety of cations *in situ*, we employed a force-mediated adhesion assay similar to Garcia and coworkers [15] or high level of uniform shear through a parallel-plate chamber [24], but in contrast to their methods, we employed longer culture times and a wider range of cation conditions, encompassing *in vivo* concentrations [23], [25], which we believe may subject integrins to force in a more biomimetic setting. By manipulating cation concentrations only during 5 minute application of shear, we found significant differences in cell attachment strength that showed a dependence on both cation concentration and cation type (i.e. Mg^2+^ or Ca^2+^). Furthermore, we demonstrate that attachment strength is drastically influenced by mechanisms of cell detachment, which are integrin-specific and differentially regulated by cations. As a consequence, our results offer alternate explanations for apparent attachment strength.

## Results

### Cations Competitively Interact to Modulate Integrin Function and Focal Adhesion Assembly under Applied Shear

**Figure 1:**
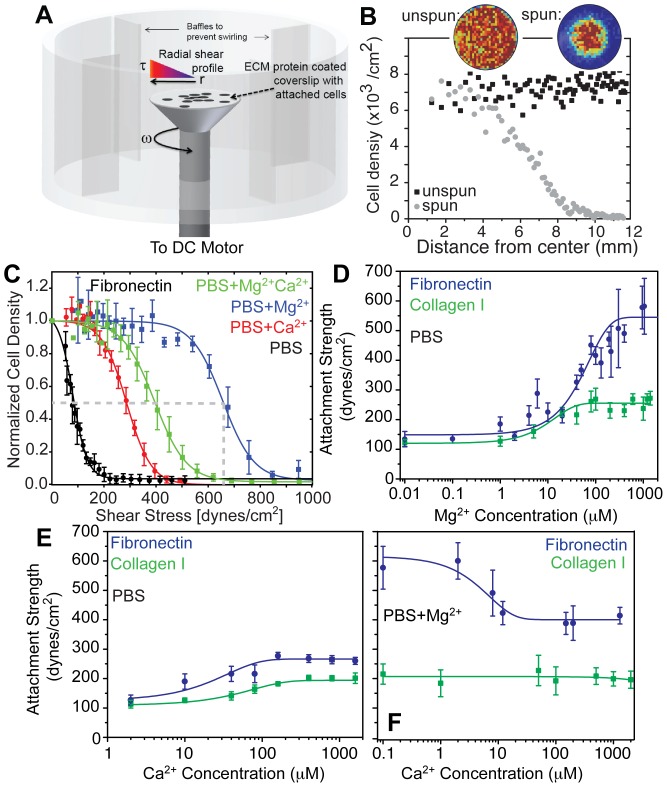
Fibroblast adhesion strength is ligand- and cation-dependent. (A) Illustration of the Spinning Disc device with cells attached to an extracellular matrix protein-coated coverslip mounted and rotating on a spinning rod in buffer. The radially-dependent shear profile is highlighted showing that cells at the center only rotate in place while those at the edge move around at a high linear velocity. (B) Plot of cell density versus coverslip position for cells that were exposed to shear (spun, gray circles) or were not (unspun, black squares). Inset images show heat maps of cell density for the indicated conditions. (C) Normalized 3T3 cell density was plotted vs. applied shear for cells with or without 0.5 mM Mg^2+^ and with or without 1 mM Ca^2+^ during the 5 min of application of shear as indicated. Note that each representative curve represents thousands of cells bound at set radial distances with data expressed as mean ± standard deviation. (D) Adhesion strength, T_50_ (measured in dynes/cm^2^), shown for 3T3 cells on fibronectin- (blue) and type I collagen-coated substrates (green) in absence of calcium but in the presence of 0.01–1000 µM Mg^2+^. Data are fit by sigmoidal curves. (E) Adhesion strength, T_50_ (measured in dynes/cm^2^), shown for 3T3 cells on fibronectin- (blue) and type I collagen-coated substrates (green) in the presence of 1–1000 µM Ca^2+^ without Mg^2+^ present. Data are fit by sigmoidal curves. (F) While keeping Mg^2+^ constant at 0.5 mM, adhesion strength was measured as a function of Ca^2+^ for both fibronectin- (blue) and type I collagen-coated substrates (green). Note that each data point in panels D-F represents triplicate experiments of thousands of cells from a coverslip exposed to a radial shear gradient. Data is expressed as mean ± standard deviation of T_50_ for each shear test at the indicated cation condition.

Fibroblasts live in many niche with different extracellular matrix (ECM) proteins and cations; to understand the extent to which fibroblast adhesion is affected by these niche conditions, murine NIH3T3 fibroblasts were cultured on fibronectin for 24 hours using standard DMEM media (Table S1) before their adhesion strength was tested by a spinning disc assay [16] (Figure 1A-B) under defined cation and matrix conditions. Fibroblast adhesion versus applied shear was plotted for cells with 0.5 mM magnesium (PBS+Mg^2+^), 1 mM calcium (PBS+Ca^2+^), and both (PBS+Mg^2+^Ca^2+^), as well as without any cation (PBS) (Figure 1C) to determine adhesion strength, i.e. T_50_–the point at which 50% of cells remain attached (Figure 1C; dashed lines). The presence of Mg^2+^ or Ca^2+^ caused an increase in cell adhesion strength by 6-fold and 2-fold, respectively, but their combination was not additive (Figure 1C). We next determined fibroblast adhesion strength to fibronectin and type I collagen as a function of Mg^2+^ (Figure 1D) or Ca^2+^ (Figure 1E) concentrations. For both matrix ligands, an increase in cation concentration caused a sigmoidal increase in adhesion strength over similar cation concentrations; however, the increase was more pronounced for fibroblasts on fibronectin above 0.1 mM Mg^2+^. Ca^2+^ has been suggested as a competitive inhibitor for initial cell attachment in the presence of both Ca^2+^ and Mg^2+^ [2], and thus adhesion strength was measured as a function of varying Ca^2+^ while Mg^2+^ was kept constant (i.e. PBS+Mg^2+^+ variable Ca^2+^; Figure 1F). Ca^2+^ in excess of 1 µM reduced adhesion strength, suggesting that it can also act as a competitive inhibitor for well-spread cells which is consistent with lower α_5_β_1_ integrin affinity in presence of Ca^2+^ [26]. Since all cells were cultured in media containing Mg^2+^ and Ca^2+^ until immediately before applying shear, these data suggest a rapid influence on integrin affinity *in situ*.

**Figure 2:**
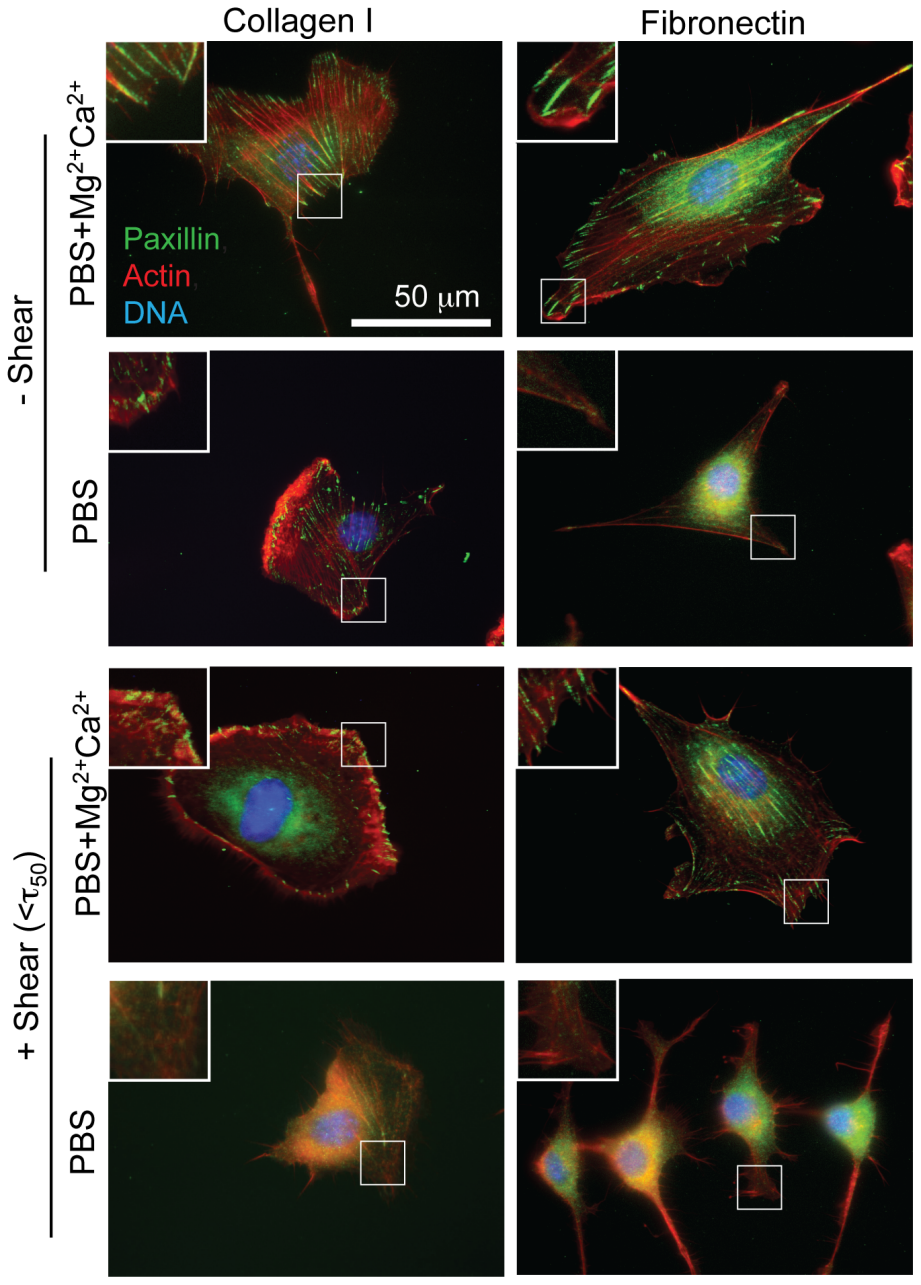
Shear- and Cation-induced Focal Adhesion (FA) Disassembly. The FA protein paxillin is displayed in green, the actin cytoskeleton in red, and the nucleus (DNA) in blue. Images were taken after 24 hrs of culture on the indicated substrate and then 5 min exposure to the indicated conditions. Inset images are shown from regions outlined in white. “+ Shear” indicates cells exposed to shear below T_50_.

Cells must transduce shear from integrins to focal adhesions (FAs), and while the above data suggest integrin modulation, they may also change adhesion strength via differential FA assembly. On both type I collagen and fibronectin substrates, fibroblasts were well spread and formed robust FAs in cultures where Ca^2+^ and Mg^2+^ were both continuously present (Figure 2, top; Figure S1, red and green); their removal alone did not affect FAs on type I collagen (Figure S1, blue) but resulted in reduced cell and FA sizes on fibronectin (Figure 2, second row; Figure S1, teal). When in the presence of shear (e.g.<T_50_) and cations, FAs were maintained (Figure 2, third row). Cell area, FA area, and FA density also did not change significantly for most comparisons with ligand-matched cells cultured with cations but absent shear (Figure S1, red vs. pink and green vs. yellow). The removal of cations during shear exposure, however, induced FA disassembly and cytoskeletal remodeling on both type I collagen and fibronectin (Figure 2, bottom), though this was more pronounced on fibronectin (Figure S1, green vs. purple). These data suggest that at low cation concentrations, applied shear can amplify FA disassembly and cytoskeletal remodeling, whereas the presence of cations can prevent this process. These data also suggest that cation driven focal adhesion changes may lead to differences in cell detachment.

### Cations Differently Modulate Cytoskeletal Detachment Mechanisms

**Figure 3:**
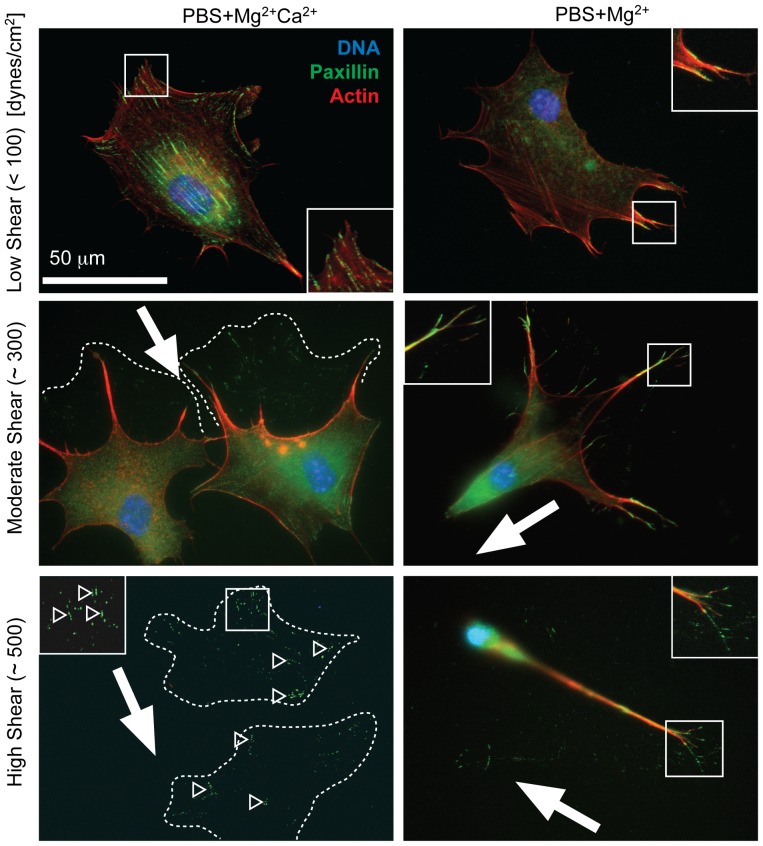
Shear-induced Cell Remodeling. 3T3 fibroblasts are shown under the indicated cation and shear conditions. The shear direction in each image is indicated by a white arrow. Images show paxillin in green, the actin cytoskeleton in red, and the nucleus (DNA) in blue. The approximate pre-shear cell area is indicated by white dashed lines as determined from the focal adhesions that remained on the substrate, which are indicated by open arrowheads. The bottom left image was contrast-enhanced 2-fold to better visualize the focal adhesions that remained on the substrate. Inset images are shown from regions outlined in white.

Although spread cell detachment has been predicted to occur via peeling [12], this process may occur quickly enough to appear binary as previously described under shear in PBS+Mg^2+^Ca^2+^ media [27]. We examined this process in greater detail at defined shear, i.e. low (<100 dynes/cm^2^), medium (∼300 dynes/cm^2^), and high (∼500 dynes/cm^2^), and we observe significant cation-dependent differences in cell morphologies (Figure 3). In PBS+Mg^2+^ media, cells exposed to high shear appeared to have aligned in the direction of applied shear (Figure 3, bottom right), which could decrease cell cross-sectional area and thus reduce their shear stress. To test this, we analyzed cell alignment and aspect ratio for cells subjected to high shear in PBS+Mg^2+^ and PBS+Mg^2+^Ca^2+^ (Figure S2). At high shear, cells in PBS+Mg^2+^ but not PBS+Mg^2+^Ca^2+^ aligned with the direction of shear, i.e. “aligned cells” (Figure S2B). Similarly, only cells in PBS+Mg^2+^ exhibited increased aspect ratio (Figure S2C); similar cell density and shear dependent aspect ratio indicate that shear alignment is the result of a morphological change instead of selection for aligned cells (Figure S2B-C). To further determine if alignment was the result of morphological changes, video imaging during shear in PBS+Mg^2+^-treated cells was used and demonstrated cell alignment via morphological changes occurred gradually and piecewise during shear exposure (Movie S1).

In PBS+Mg^2+^Ca^2+^ media, cells did not align with shear, and many cells detached piecewise where they existed temporarily in a partially detached state, i.e. “remodeled cells” (Movie S2). Paxillin-containing puncta were clearly visible around partially detached, “remodeled” cells and are highlighted in Figure 3 and Figure S3 with dashed lines drawn around the puncta to indicate the approximate boundary of the cell. Regions where completely detached cells likely were attached could also be observed from paxillin-containing puncta (Figure 3, bottom left; Figure S3). Importantly, we only observed alignment on fibronectin and PBS+Mg^2+^ and not in other conditions, e.g. PBS+Ca^2+^ cells (Figure S3, left) and PBS+Mg^2+^ cells cultured on collagen I (Figure S3, right), where detachment occurred through other modes including remodeling with paxillin puncta remaining on the substrate. It should be noted that some [28], [29] but not all [12], [30] quantitative assessments of adhesion have detected cell components that remain attached after shear. Here paxillin puncta were not present on substrates in absence of cations and were minimally present around cells when subjected to shear below T_50_ (e.g. Figures 2 vs. Figure 3 at low shear); these data suggest that detachment is piecewise and that partially detached cells may leave adhesive proteins bound to the substrate under the appropriate shear and cation conditions.

**Figure 4:**
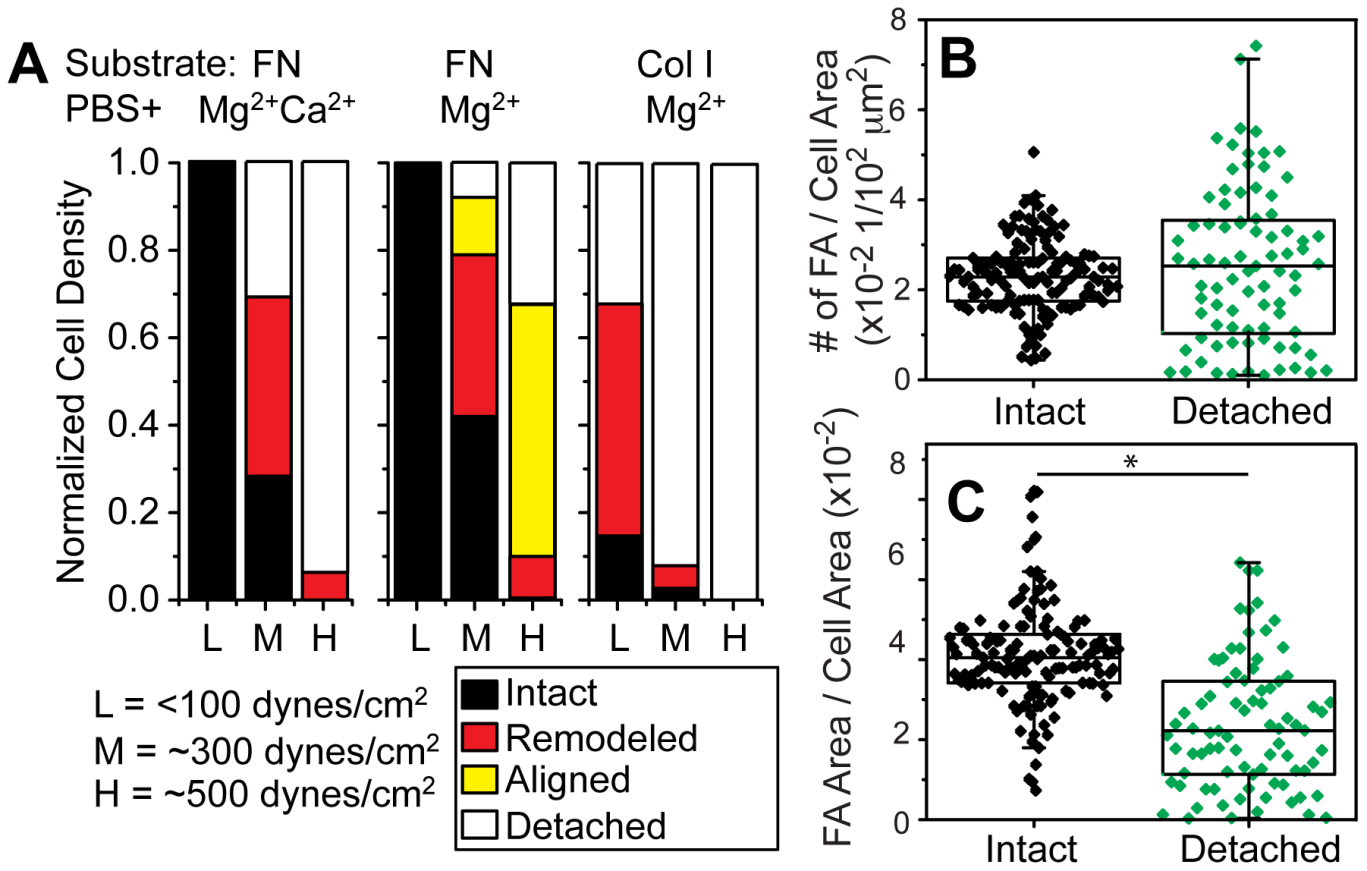
Analysis of Cell Detachment Mechanisms. (A) The occurrence of different detachment modes are indicated for cells treated with the indicated matrix ligands, cation concentrations, and shear. Cells were characterized as: 1) “Remodeled”, defined as nucleus present but visible cell deformation with paxillin puncta surrounding the cell, e.g. center and lower right images of Figure 3 (please note that no differentiation was made between deformed cells and aligned cells as the differences are gradual until full alignment is reached), 2) “Aligned”, defined as cells aligned in the direction of shear determined by a combination of cell aspect ratio and direction of major axis, 3) “Detached”, determined from the density difference between other conditions and unspun cells cells. For each condition the same surface area was scanned and analyzed, where the low shear region, e.g.<100 dynes/cm^2^, contained at least 100 cells. Focal adhesion density, (B) based on number of discrete adhesions or (C) on the area of those adhesions versus cell area, was determined for intact cells or detached cells that left behind paxillin-containing puncta. For detached cells, the area was determined by the maximum extent of the puncta. For each condition, at least 80 intact cells from triplicate experiments were scanned and analyzed. *p<0.05.

To better describe the frequency of remodeled and aligned cell fractions in a variety of niche conditions, fibroblasts were subjected to defined shear stresses of <100 dynes/cm^2^, ∼300 dynes/cm^2^, and ∼500 dynes/cm^2^ in the conditions indicated in Figure 4A. Shear below 100 dynes/cm^2^ in most cases did not result in significant detachment, but application of ∼300 dynes/cm^2^ in PBS+Mg^2+^Ca^2+^ media on fibronectin substrates caused ∼40% of cells to partially peel and ∼30% of cells to completely detach from the substrate; higher shear caused nearly all cells to detach (Figure 4A, left). Puncta density on the coverslip was similar to the density of intact cells (Figure 4B), implying that most adhesions ruptured despite a drop in the area of these puncta (Figure 4C). However in the absence of Ca^2+^ and shear at or exceeding 300 dynes/cm^2^, most cells remained attached with cell alignment increasing with shear magnitude (Figure 4A, middle). With these drastic morphological changes, it was not clear if aligned cells remained viable, but time lapse imaging of aligned cells post shear indicate recovery and spreading within 1 hour after shear exposure (Figure S2D). Cell alignment was not observed on type I Collagen substrates in PBS+Mg^2+^ media, but rather significant peeling occurred at low shear (Figure 4A, right). These data imply that cell alignment with shear is substrate dependent and may contribute to the heterogeneity observed in cell detachment, i.e. the width of the sigmoidal curve in Figure 1.

### Shear-Induced Remodeling and its Cytoskeletal and Integrin Regulation is Ubiquitous

**Figure 5:**
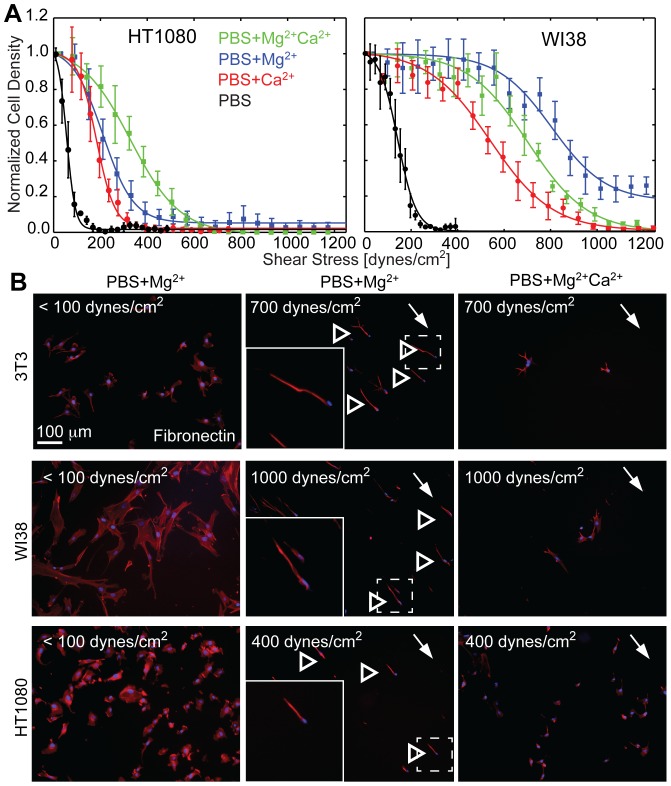
Human Fibroblasts and Fibrosarcoma Cells bound to Fibronectin also Align in Defined Cation Conditions. (A) Cell density of human fibroblasts (WI38) and human fibrosarcoma cells (HT1080) attached to fibronectin were plotted as a function of the applied shear stress. Cation conditions are indicated. Note that each representative curve represents thousands of cells grouped together at set radial distances with data expressed as mean ± standard deviation. (B) For the indicated cation and shear stress conditions, fluorescent images of 3T3 fibroblasts, WI38 fibroblasts, and HT1080 fibrosarcoma cells are shown. Cells were stained for DNA (blue) and actin (red). The direction of shear is indicated for cells subjected to higher stress (center and right images). Some aligned cells are indicated by open arrowheads. Dashed lines indicate the regions of inset images, which are outlined in white.

Many population-based adhesion studies have been performed on other cell types and species, e.g. human fibrosarcoma [31], human glioma cell lines [32], and bovine chondrocytes among others [33], but few have examined the impact of cation conditions on cell remodeling. Therefore, we next determined how ubiquitous these unique detachment modes and cation type dependencies were in shear-induced detachment of HT1080 human fibrosarcoma cells and WI38 human fibroblasts. Human fibrosarcoma cells are most weakly attached in PBS and PBS+Ca^2+^ media, but unlike murine fibroblasts, HT1080 cells are most strongly adherent in PBS+Mg^2+^Ca^2+^ media. As with murine fibroblasts, human fibroblasts attached most strongly to fibronectin substrates in PBS+Mg^2+^, followed by PBS+Mg^2+^Ca^2+^, PBS+Ca^2+^, and PBS media (Figure 5A). Unlike murine fibroblasts, ∼20% of WI38 cells remained adherent even at the highest applied shear in PBS+Mg^2+^ media (Figure 5A, blue), and an examination of cell morphology post-shear showed significant alignment in the direction of shear, as previously seen with 3T3 cells. Alignment was also observed for HT1080 cells in PBS+Mg^2+^ media, albeit with fewer cells remaining and at lower shear (Figure 5B, open arrowheads). As with 3T3 cells alignment, we did not observe alignment of HT1080 and WI38 in any condition other than PBS+Mg^2+^ media and fibronectin. A direct comparison of the cation-dependent attachment strength of all 3 cell lines on Fibronectin vs. Collagen type 1 shows that drastic attachment differences appear only on Fibronectin but not on Collagen (Figure S4A and B). As only HT1080 cells on Fibronectin adhere stronger when both cations are combined (PBS+Mg^2+^Ca^2+^ media), we analyzed their cation concentration-dependent attachment strength behavior in more detail (Figure S4C-E). In the presence of 500 µM Mg^2+^, the addition of ∼100 µM Ca^2+^ was sufficient to increase the attachment strength of HT1080 cells independent of matrix condition; at 1 mM Ca^2+^ attachment strength is increased by ∼50% on fibronectin but not on collagen (Figure S4E).

**Figure 6:**
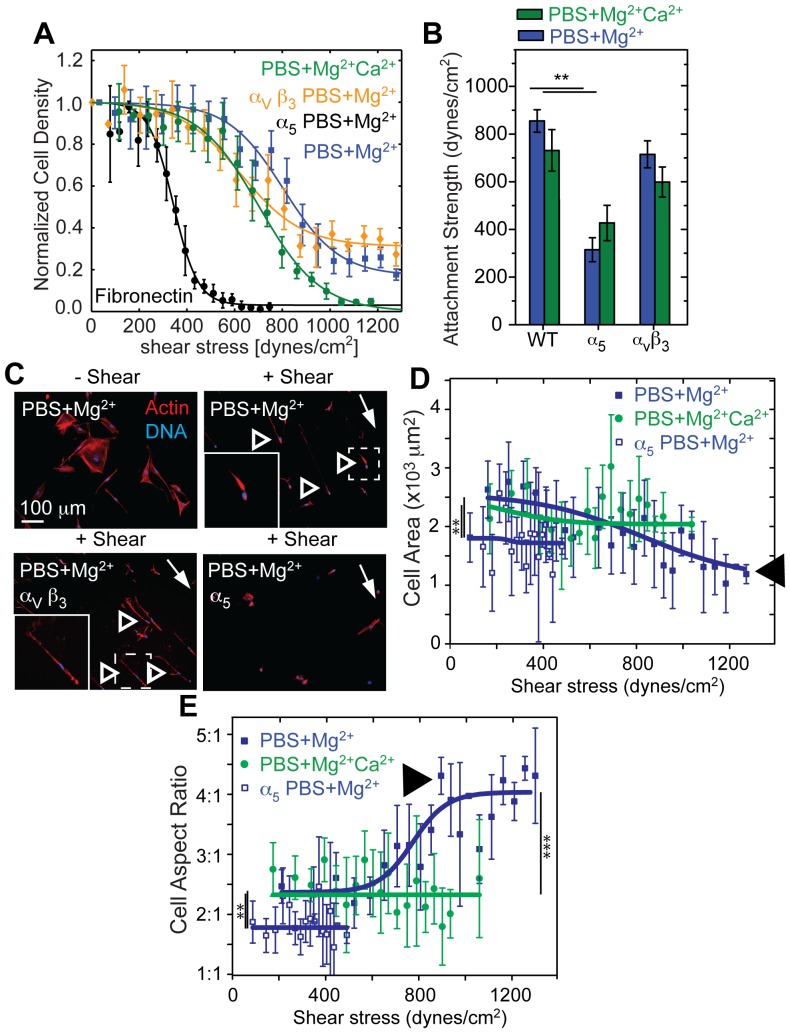
α_5_ Integrins Regulate Cell Remodeling under Shear. (A) WI38 fibroblast adhesion strength was tested under the indicated conditions. Note that each representative curve represents thousands of cells grouped together at set radial distances with data expressed as mean ± standard deviation. (B) Average adhesion strength (T_50_) for WI38 fibroblasts for the indicated conditions from triplicate experiments. (C) Fluorescent images of WI38 cells stained pre- or post-shear for nuclei (DNA; blue) and actin (red). Shear direction is indicated by a white arrow and remodeled cells are indicated by open arrowheads. Dashed lines indicate the regions of inset images, which are outlined in white. (D) Cell area vs shear stress of WI38 cells is plotted. Only when engaged with α_5_ integrins and in absence of Ca^2+^ did cell area decrease significantly with increasing shear (black arrow head). Please note that cells with blocked α_5_ integrins or in presence of Ca^2+^ detach at lower shear. (E) Cell aspect ratio vs shear stress of WI38 cells is plotted. Only when engaged with α_5_ integrins and in absence of Ca^2+^ did cell aspect ratio significantly increase with increasing shear (black arrow head). For panels D and E, statistical tests were performed between the different conditions indicated as a function of shear using non-parametric Kruskal-Wallis analysis of variance. Note that data was grouped together at set radial distances expressed as mean ± standard deviation for each curve and represents thousands of cells. Statistical analysis for all other panels were performed as indicated in the methods section. *p<0.05, ** p<0.01, ****p<0.0001.

**Figure 7:**
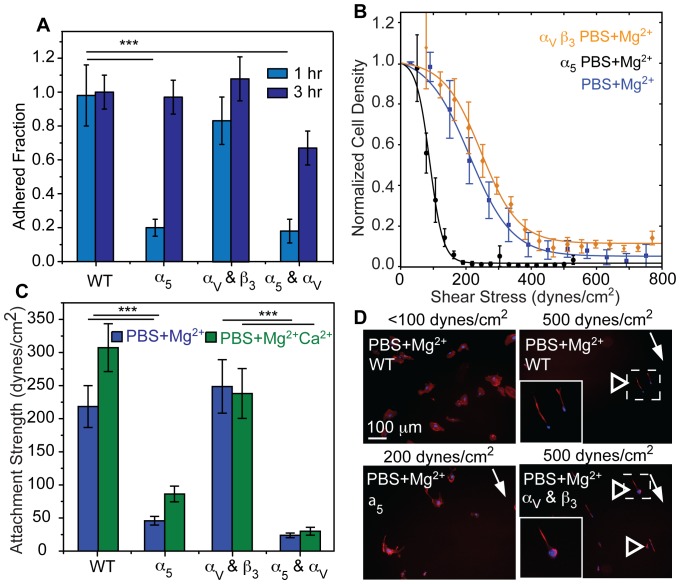
α_5_ Integrins Regulate HT1080 Fibrosarcoma Cell Remodeling Under Shear. (A) Coverslips were washed at the indicated times to eliminate unbound HT1080 fibrosarcoma cells, and the remaining cell density, normalized to untreated (WT) and unwashed controls at the indicated time points, is plotted for the indicated treatments. (B) Cell attachment strength to fibronectin was tested under the indicated conditions. Note that each representative curve represents thousands of cells grouped at set radial distances with data expressed as mean ± standard deviation. (C) Average attachment strength (T_50_) for cells in the indicated conditions. (D) Fluorescent images of cells stained after application of the indicated shear for nuclei (DNA; blue) and actin (red). A white arrow indicates shear direction and open arrowheads indicate remodeled cells. Dashed lines indicate the regions of inset images, which are outlined in white. Note that all statistical analyses used non-parametric Kruskal-Wallis analysis of variance from triplicate experiments. ***p<0.001.

Ligand-specific differences modulate remodeling, but it is unclear if alignment is specific to either α_5_β_1_ or α_v_β_3_ integrins. In conditions where alignment was most prevalent, i.e. WI38 fibroblasts on fibronectin substrates in PBS+Mg^2+^ media, blocking the function of α_5_ but not α_v_β_3_ integrin reduced cell area and total FA area but not the number of FAs normalized by the reduced cell area or the FA density (Figure S5). As a consequence of smaller but still numerous adhesions, α_5_ blocking reduced adhesion strength by more than half (Figure 6A-B), consistent with previous reports [34], and ablated the elongated phenotype characteristic of alignment under applied shear (Figure 6C). As with 3T3 cells (Figure 3), alignment of WI38 cells was shear and α_5_ integrin dependent: cell area decreased while alignment increased in PBS+Mg^2+^ (Figure 6D-E, arrowheads). Blocking the function of α_5_ but not α_v_β_3_ integrin in HT1080 cells resulted in reduced cell area, total FA area, and number of FAs (Figure S6A-G). Blocking also lead to defects in initial attachment as well as adhesion strength and alignment (Figure 7) as with WI38 cells. In contrast to WI38 cells though, function blocking α_5_ integrin did cause a more elongated cell phenotype (Figure S6H). With a marked reduction of the fraction of cells at highest shear and no alignment during α_5_ blockage, HT1080 cells rely on α_5_ integrin function for their alignment, as in the case of WI38 cells. Function blocking α_v_β_3_ integrin had little effort in either HT1080 or WI38 cell morphology, adhesion strength, or alignment (Figures 6, 7, S5 and S6) consistent with α_5_ integrin supporting the majority of force transmission. However, it is important to note that both cell types, WI38 and HT180, expressed similar amounts of α_5_ and α_V_ integrin (Figure S4I-K). Thus, these data show that the adhesive engagement by α_5_ integrin is a necessary, but not sufficient, condition for alignment.

## Discussion

### Cation Dependence of Cell Adhesion Strength

Cation-specific differences in initial attachment have been classically observed across a variety of cell types [35], [36] where attachment is faster and stronger in the presence of Mg^2+^ versus Ca^2+^ [37]–[39]. To understand how a variety of cations modulate integrin function after establishment of adhesion *in situ*, we employed a force-mediated adhesion assay after the establishment of a well spread morphology and over a range of cation conditions encompassing the *in vivo* ionized cation concentrations [21]–[23], [25], which may subject integrins to a more physiological setting. We found that across all cell types, removal of cations resulted in a significant loss of adhesion strength. Murine and human fibroblast adhesion strength was higher when Mg^2+^ is present but not Ca^2+^ in agreement with single-molecule experiments on α_5_β_1_ integrin [26]. In contrast, human HT1080 fibrosarcoma adhesion strength was highest when both Mg^2+^ and Ca^2+^ were present to bind to fibronectin. Generally though, HT1080 fibrosarcoma cells were the most sensitive to cation removal, and since tumor cation levels can be higher than healthy stroma [23], adhesion regulation may help control tumor cell dissemination. Our data also suggest that these adhesion strength differences cannot be explained by differences in integrin expression or type; rather, these data imply that there must be other differences between cell types that regulate the response of integrin affinity to cations might be *in situ*. Finally, these data highlight the need to mimic the cation concentrations and composition, e.g. Mg^2+^ versus Ca^2+^, for a given niche as cell type-specific adhesion strength can be dramatically affected.

### Stepwise Detachment and Remodeling

**Figure 8:**
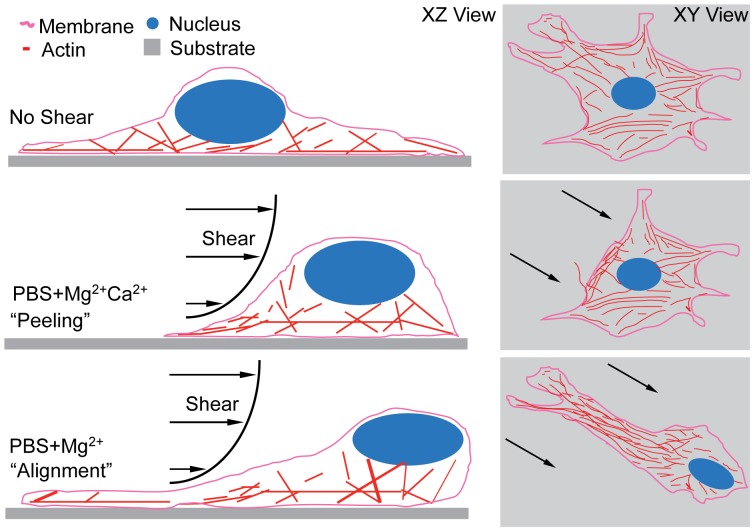
Cell Detachment Mechanisms. Schematic of XZ (left) and XY (right) images depicts the possible cell detachment mechanisms for the given cation conditions, suggesting that a cell (top) with PBS+Mg^2+^Ca^2+^ media undergoes detachment by piecewise peeling while a cells with PBS+Mg^2+^ media can align to the direction of applied shear (bottom).

During adhesion maturation, cells undergo a complex interplay of integrin binding, focal adhesion assembly, and cell spreading. Due to this complex organization, extracellular forces, e.g. shear stress, are heterogeneously transmitted to the FAs. With the highest forces acting on the cell periphery, detachment has been suggested to happen by a peeling mechanism with piecewise detachment beginning at the cell periphery [12]. However as peeling has not been observed in detachment assays using fully spread cells, detachment has been suggested to occur quickly and thus described as either attached or detached, without intermediate states [15], [16], [27]. Our data suggest that many, but not all, cells detach stepwise beginning at the leading edge while undergoing cytoskeletal remodeling (Figures 3), where the stress on FAs is highest, and continuing rearward in presence of Ca^2+^ or when seeded on type I collagen (Figure 8, middle condition). Peeling occurred over minutes and was the major mechanism of detachment found with every cation and substrate condition after 5 minutes of shear exposure close to T_50_ (Figure 4; Movie S1-2). We observed a wide variety in peeling onset and duration ranging from immediate detachment to remodeling slowly during application of shear to no remodeling at all (Movie S1-2 and further observations). While the reason for heterogeneity remains unclear, it offers an additional mechanism for the observed width in cell density vs shear plots which has previously been explained only by factors as cell cycle state, size, and FA density [12], [16].

### Cell Alignment is Cation and Integrin Type-Dependence

In stepwise detachment, cells most likely detach from FAs with highest stress first. However when cells aligned in PBS+Mg^2+^, they minimized their cross-sectional area perpendicular to the shear direction, and thus reduced the effective external force. Once fully aligned, a subset of WI38 cells remained attached up to highest applicable shear, mediated by α_5_β_1_ integrins (Figure 6A). Previous force spectroscopy measurements have shown that α_5_β_1_ integrin affinity and bond lifetime under force are higher in PBS+Mg^2+^ versus PBS+Mg^2+^Ca^2+^ media [26], and this could explain the differences in adhesion strength. It is also important to note that reduced spread area also causes reduced FA density, but the catch bond behavior of α_5_β_1_ but not α_v_β_3_ integrins and the specificity of alignment to α_5_β_1_ integrins suggest that cells may be able to sustain the higher force experienced per unit area of adhesion because of the preference for α_5_β_1_ integrins [40]. Yet the alignment of cells with shear suggests that active cell-based mechanisms may control how cells remodel and detach rather than maximal integrin-ECM bond strength. Cells have precise spatiotemporal control over adhesions and the forces they bear; FA growth and recruitment can be directionally specific in response to applied forces [41] and even adjacent focal adhesions can have different traction forces [42]. Thus, it is plausible that dynamic integrin binding, traction forces, and shear stresses can combine to preferentially break some peripheral adhesions and rigidify others to make alignment an adaptive mechanism to support higher force experienced per unit area of adhesion. Furthermore, HT1080 cells express a similar amount α_5_ integrins compared to WI38 cells (Figure S6I-K), but are markedly less able to align. These data would imply that α_5_β_1_ integrins are necessary but not sufficient for alignment as the dynamics of the cytoskeleton can be modulated by cation-dependent integrin state [43]. While this could act as another regulator of the decision between peeling and aligning, the cytoskeletal underpinnings of these two detachment mechanisms are beyond the scope of the current efforts.

In summary, the data presented here demonstrate how local niche conditions regulate adhesion strength rather than describing detachment mechanisms exclusively in terms of the integrin-ECM interface. It also suggests additional mechanisms of how integrins, FAs, and the cytoskeleton act together to permit focal adhesion-mediated cell detachment in a more dynamic manner than was previously appreciated. Given the wide variety of niches in which adherent cell types inhabit, these data suggest the need to re-examine of the mechanisms of cell adhesion, especially at physiologically relevant cation concentrations.

## Materials and Methods

### Cell Culture

Mouse NIH 3T3 fibroblast cells, human WI38 fibroblast cells, and human HT1080 fibrosarcoma cells were obtained from ATCC and cultured in their respective media listed in Table S1, noting typical formulations from Life Technologies. All cells were cultured at 37°C in a humidified incubator containing 5% carbon dioxide. Function blocking antibodies, BIIG2 (a gift from Caroline Damsky, University of California, San Francisco, CA), 9H5 (a gift from David Strom, Des Moines University, IA) or LM609 (Millipore), were added at 10 µg/mL concentration to cells suspended in 200 µL culture media 45 minutes prior to plating. Unless otherwise noted, cell culture products purchased were from Life Technologies (Carlsbad, CA).

### Cell Adhesion Strength and Wash Assay

25-mm glass coverslips (Fisher Scientific, St. Louis, MO) were sonicated with ethanol and pure water before being used for incubation of 10 µg/mL human fibronectin (isolated from serum [44]) or 20 µg/ml type I collagen (rat tail, BD Bioscience) for 60 min at room temperature. Under regular conditions cells were allowed to attach for 24 hrs at 37°C and 5% CO_2_ using the media described in Table S1; when integrin function blocked, cells were allowed to adhere for 2-3 hours. The coverslips were then mounted on a custom-built spinning disc device and dipped into the temperature-controlled spinning buffer (37°C). As spinning buffer, PBS without magnesium and calcium or with 0.5 mM MgCl_2_ and 1 mM CaCl_2_ (Cellgro, Manassas, VA) was used. It is to be noted that commercial PBS for cell culture is usually cation free. All spinning buffers contained 4.5 mg/mL Dextrose. Once immersed into the spinning buffer, coverslips were spun for 5 min at defined angular velocities and fixed with 3.7% formaldehyde immediately after spinning. For wash assays, the coverslips were dipped 3 times in the spinning buffer and then fixed.

### Quantification of Adhesion Strength

Shear stress τ by radial fluid motion over the surface of the coverslip was calculated according to [16] such that:
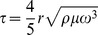
(Eqn1)where r is the radial position from the center of the disk, ρ is the buffer density, μ is the buffer viscosity and ω is the rotational speed. To obtain quantitative information of adhesion strength, whole 25 mm coverslips were imaged at 10x magnification on a Nikon Ti-S microscope (∼1000 individual images stitched together with Metamorph 7.6 software and custom macros) and analyzed using a custom written MATLAB program. In brief, the user defines the outer circle of the coverslip from a stitched overview image and the software then finds the position of each nucleus relative to the center of the coverslip. Cell densities, normalized to the densities of unspun coverslips, as a function of radial position and subsequently shear, are stored and combined with other measurements e.g. those obtained at different RPMs. A sigmoidal fit is used to quantify values of adhesion strength and determine the statistical error of the fit. Additionally, to determine cell alignment, cell morphology was analyzed similarly as a function of shear for each cell when stained for actin cytoskeleton.

### Immunofluorescence staining and Focal Adhesion analysis

Fixed cells were incubated for 10 min with 0.25% Triton X-100 followed by 1% albumin overnight at 4°C for blocking. Primary paxillin antibody (1∶2000, ab32084, Abcam) was applied for 2 hours at room temperature, and then a secondary AlexaFluor 488-conjugated antibody (1∶2000, Invitrogen) was applied for 1 hour or rhodamine phalloidin (1∶2000 Invitrogen) and Hoechst 33342 (3.2 µM, Invitrogen) for 30 min at room temperature. The cells were subsequently mounted with Fluoromount-G (Southern Biotech, Birmingham, AL). All buffers used contained 1 mM MgCl_2_. The samples were imaged by using a CARV II confocal (BD Biosciences) Nikon Eclipse Ti-S microscope equipped with a motorized, programmable stage using a Cool-Snap HQ camera (Photometrics) and controlled by Metamorph 7.6 (Molecular Devices). A custom-written MATLAB (Mathworks) program was used to quantify cell area and focal adhesion number and size.

### Linear Shear Stress Flow Chamber

A custom-build flow chamber was used for live cell imaging [24]. To achieve shear stress of up to 400 dynes/cm^2^, the channel width was reduced to 2 mm and its height to 80 µm. Up to 7.5 ml of spinning buffer were pumped per minute through the chamber using a syringe pump. Video images were captured at 10x resolution during application of shear.

### FACS Analysis

WI38 and HT1080 cells were detached from fibronectin-treated coverslips by incubation for 5-10 min with PBS without cations at 37°C and gentle pipetting. After resuspension in flow cytometry buffer (DPBS, 2.5% goat serum, 1 mM EDTA, pH 7.4), the cells were incubated with fluorescent-conjugated antibodies against CD49e (PE) and CD51 (FITC) (Biolegend) for 30 min on ice. Cells were analyzed using a FACScan Flow Cytometer (BD Biosciences).

### Statistical Analysis

Non-parametric Kruskal-Wallis analysis of variance tests were used for all statistical analysis. All data in shear plots are expressed as mean ± standard deviation. Data in box plots are expressed as mean and the 10^th^ and 90^th^ percentile. All experiments were performed at least in triplicate and analyses represent hundreds of cells per condition.

## Supporting Information

Figure S1 **Quantification of Cell and FA Parameters for NIH 3T3 Cells Under Varying Cation, Matrix, and Shear Conditions.** (A) Cell area and focal adhesion area (C) are shown for cells with or without shear, with or without Mg^2+^ and Ca^2+^ cations, and on fibronectin (FN) or type I collagen (Col) substrates. The same combination of conditions is shown for the density of focal adhesions based on number of discrete adhesions (B) and area (D). n = 25 to 228 cells for each condition from triplicate experiments. “+ Shear” indicates cells exposed to shear below T_50_. *p<0.05, ** p<0.01.
(TIF)

Figure S2 **Quantification of Shear-induced Cell Alignment and Recovery.** (A) Representative heat map (center) of 3T3 fibroblast density after having been subjected to high shear in presence of Mg^2+^ but not Ca^2+^ to encourage remodeling. Warm and cool colors in the heat map signify high and low cell density, respectively. Fluorescent images showing DNA (blue) and actin (red) from the indicated locations demonstrate alignment with the shear angle but not radial position. The white and yellow arrows on the images indicate direction of disc motion and the direction of the cell's major axis, respectively. Alignment offset between the two angles is indicated as δ. (B) Quantification of cell alignment from the selected regions in panel A is plotted using a kernel density function for the indicated media conditions to indicate average cell orientation to the shear direction. Note that there is no statistical difference for data at different angular positions for the same radial position. (C) For the same selected regions and media conditions, cell aspect ratio was normalized by cell densities and graphed using a kernel density function. (D) Selected images from time-lapse video microscopy show that fibroblasts on fibronectin substrates in PBS+Mg^2+^ media have elongated and aligned immediately after shear (time  =  00:00 but can re-spread after shear. Arrowhead indicates a recovering fibroblast.
(TIF)

Figure S3 **Shear-induced Cell Remodeling for Non-Aligning Conditions.** 3T3 fibroblasts are shown under the indicated cation and ligand conditions. Shear direction in each image is indicated by a white arrow. Images show paxillin in green, the actin cytoskeleton in red, and the nucleus (DNA) in blue. The approximate pre-shear cell area is indicated by white dashed lines as determined from the focal adhesions that remained on the substrate.
(TIF)

Figure S4 **Quantification of Shear-induced Cell Remodeling for Non-Aligning Conditions.** (A-B) Attachment strength of 3T3, WI38 and HT1080 cells under the indicated cation and ligand conditions. (C) Adhesion strength, T_50_ (measured in dynes/cm^2^), for HT1080 cells on fibronectin- (blue) and type I collagen-coated substrates (green) in absence of calcium but in the presence of 0.01–1000 µM Mg^2+^. Data are fit by sigmoidal curves. (D) Adhesion strength, T_50_ (measured in dynes/cm^2^), for HT1080 cells on fibronectin- (blue) and type I collagen-coated substrates (green) in the presence of 1–1000 µM Ca^2+^ without Mg^2+^ present. Data are fit by sigmoidal curves. (E) While keeping Mg^2+^ constant at 0.5 mM, adhesion strength was measured as a function of Ca^2+^ for both fibronectin- (blue) and type I collagen-coated substrates (green).
(TIF)

Figure S5 **Blocking α_5_ but not α_v_ Integrin Function without Shear in Magnesium-containing Media alters Attachment of WI38 Fibroblasts.** (A-C) 60x fluorescence images of WI38 fibroblasts 2 hours post-seeding on fibronectin showing paxillin (green), actin (red) and DNA (blue). Inset images are shown from regions outlined in white. Cells were treated with the indicated conditions: (A) WT, (B) blocking α_5_ integrins, and (C) blocking β_3_ integrins. (D-G) Quantification of indicated morphological and FA parameters for the same conditions in panels A-C performed in triplicate. * p<0.05, *** p<0.001. 10x fluorescence images of WI38 fibroblasts, actin (red) and DNA (blue), after cyt D treatment (bottom) and without (top) as well as low (left) and high (right) application of shear. Direction of applied shear indicated by arrow.
(TIF)

Figure S6 **Blocking α_5_ but not α_v_ Integrin Function without Shear in Magnesium-containing Media for HT1080 Fibrosarcoma Cells.** (A-C) Fluorescence images of HT1080 fibrosarcoma cells 3 hours post-seeding showing paxillin (green), actin (red) and DNA (blue). Inset images are shown from regions outlined in white. Cells were treated with the indicated conditions: (A) WT, (B) blocking α_5_ integrins, and (C) blocking β_3_ integrins. (D-H) Quantification of indicated morphological and FA parameters for the same conditions in panels A-C. (I-J) Flow cytometry comparing α_5_ and α_V_ integrin expression peaks for WI38 fibroblasts and HT1080 fibrosarcoma cells. (K) Shown are ratios of integrin subtypes within a single cell type (left) and for a single integrin subtype between cell types (right). *** p<0.001, N.S.  =  not significant.
(TIF)

Table S1 **Standard media formulations for each cell type used with Dulbecco's modified Eagle's medium (DMEM) are listed.** Additional components and concentrations not specifically mentioned here are 4 mM L-glutamine, 1 mM sodium pyruvate, and 100 U/mL penicillin. The table specifically notes standard cation concentrations in commercially available solutions of DMEM and serum (column 3; [42]) and the range tested (column 4), with specific concentrations indicated in the text.
(DOCX)

Movie S1 **Cell detachment during application of Shear in PBS+Mg^2+^ conditions.** Cells cultured on fibronectin substrates were imaged for 5 minutes at 10x magnification in the linear shear stress flow chamber during application of ∼400 dynes/cm^2^ shear in presence of Mg^2+^ but not Ca^2+^.
(M4V)

Movie S2 **Cell detachment during application of Shear in PBS+Mg^2+^Ca^2+^ conditions.** Cells cultured on fibronectin were imaged for 5 minutes at 10x magnification in the linear shear stress flow chamber during application of ∼400 dynes/cm^2^ shear in presence of Mg^2+^ and Ca^2+^.
(M4V)
