# Supplementary figures and images for: Cation Type Specific Cell Remodeling Regulates Attachment Strength
Source: PLoS One. 2014 Jul 11;9(7):e102424. doi: 10.1371/journal.pone.0102424 (PMC4094514; doi:10.1371/journal.pone.0102424)

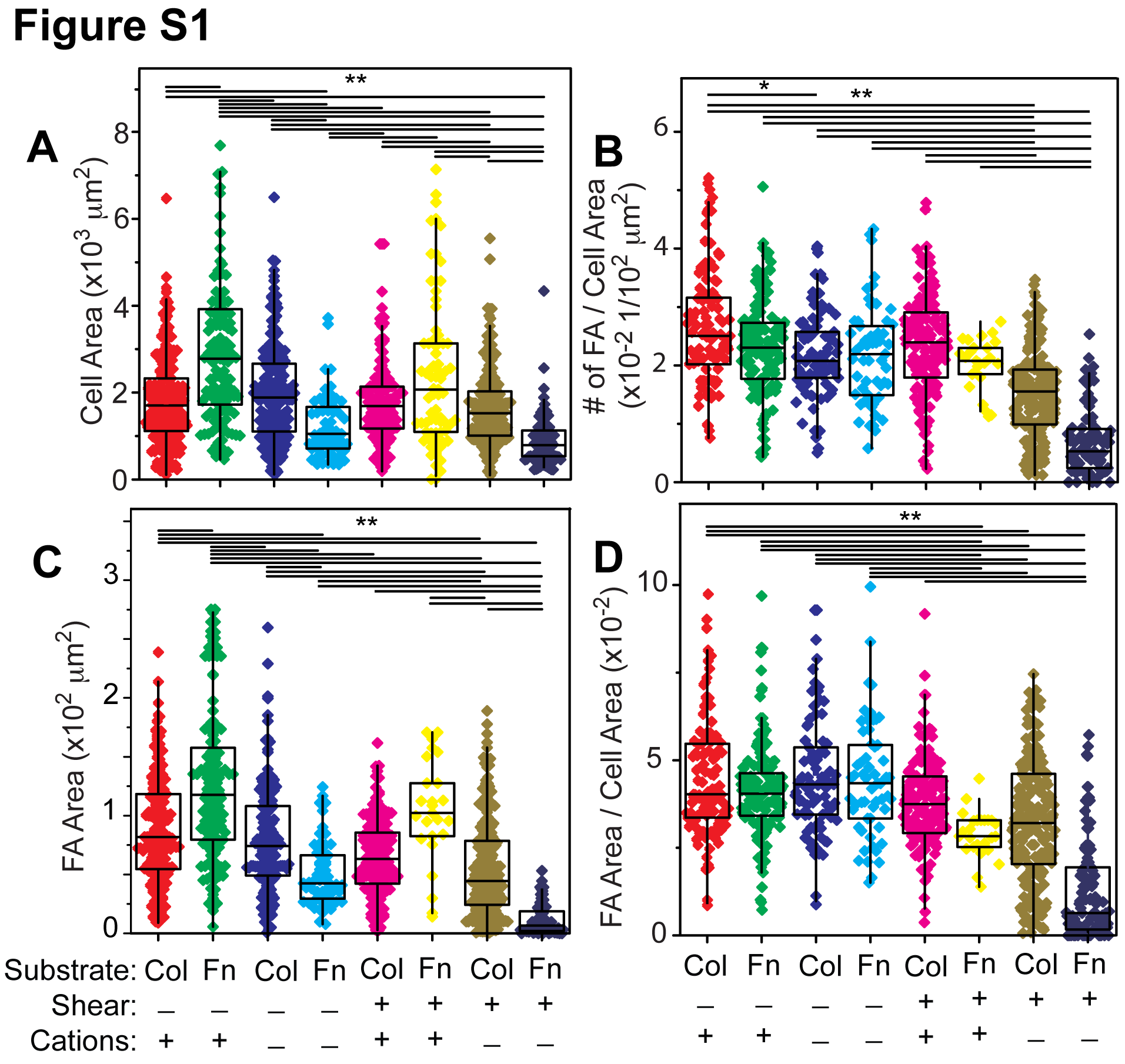

Supplement: Figure S1 — Quantification of Cell and FA Parameters for NIH 3T3 Cells Under Varying Cation, Matrix, and Shear Conditions. (A) Cell area and focal adhesion area (C) are shown for cells with or without shear, with or without Mg2+ and Ca2+ cations, and on fibronectin (FN) or type I collagen (Col) substrates. The same combination of conditions is shown for the density of focal adhesions based on number of discrete adhesions (B) and area (D). n = 25 to 228 cells for each condition from triplicate experiments. “+ Shear” indicates cells exposed to shear below T 50. *p<0.05, ** p<0.01. (TIF) [file pone.0102424.s001.tif]

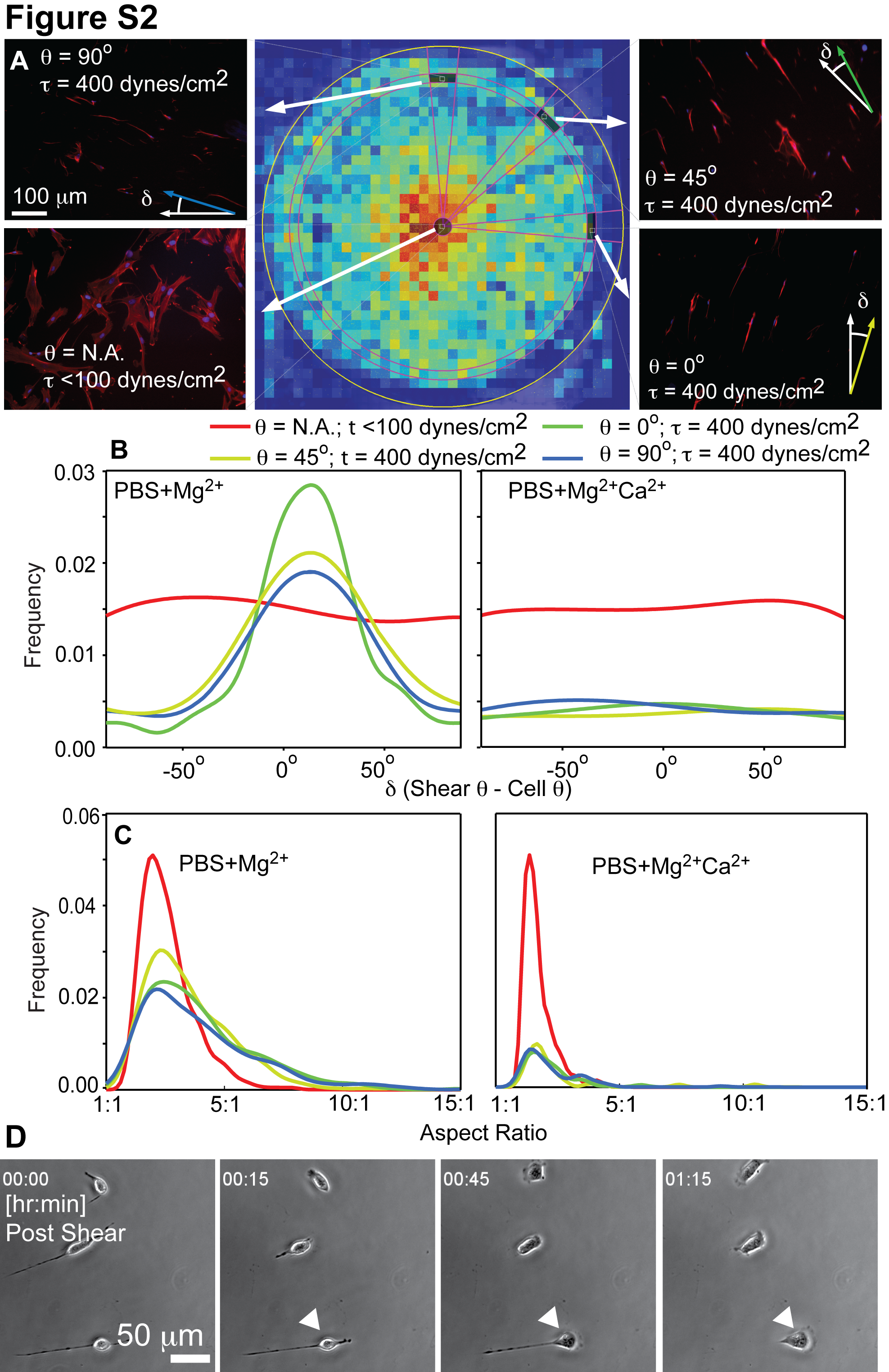

Supplement: Figure S2 — Quantification of Shear-induced Cell Alignment and Recovery. (A) Representative heat map (center) of 3T3 fibroblast density after having been subjected to high shear in presence of Mg2+ but not Ca2+ to encourage remodeling. Warm and cool colors in the heat map signify high and low cell density, respectively. Fluorescent images showing DNA (blue) and actin (red) from the indicated locations demonstrate alignment with the shear angle but not radial position. The white and yellow arrows on the images indicate direction of disc motion and the direction of the cell's major axis, respectively. Alignment offset between the two angles is indicated as δ. (B) Quantification of cell alignment from the selected regions in panel A is plotted using a kernel density function for the indicated media conditions to indicate average cell orientation to the shear direction. Note that there is no statistical difference for data at different angular positions for the same radial position. (C) For the same selected regions and media conditions, cell aspect ratio was normalized by cell densities and graphed using a kernel density function. (D) Selected images from time-lapse video microscopy show that fibroblasts on fibronectin substrates in PBS+Mg2+ media have elongated and aligned immediately after shear (time = 00:00 but can re-spread after shear. Arrowhead indicates a recovering fibroblast. (TIF) [file pone.0102424.s002.tif]

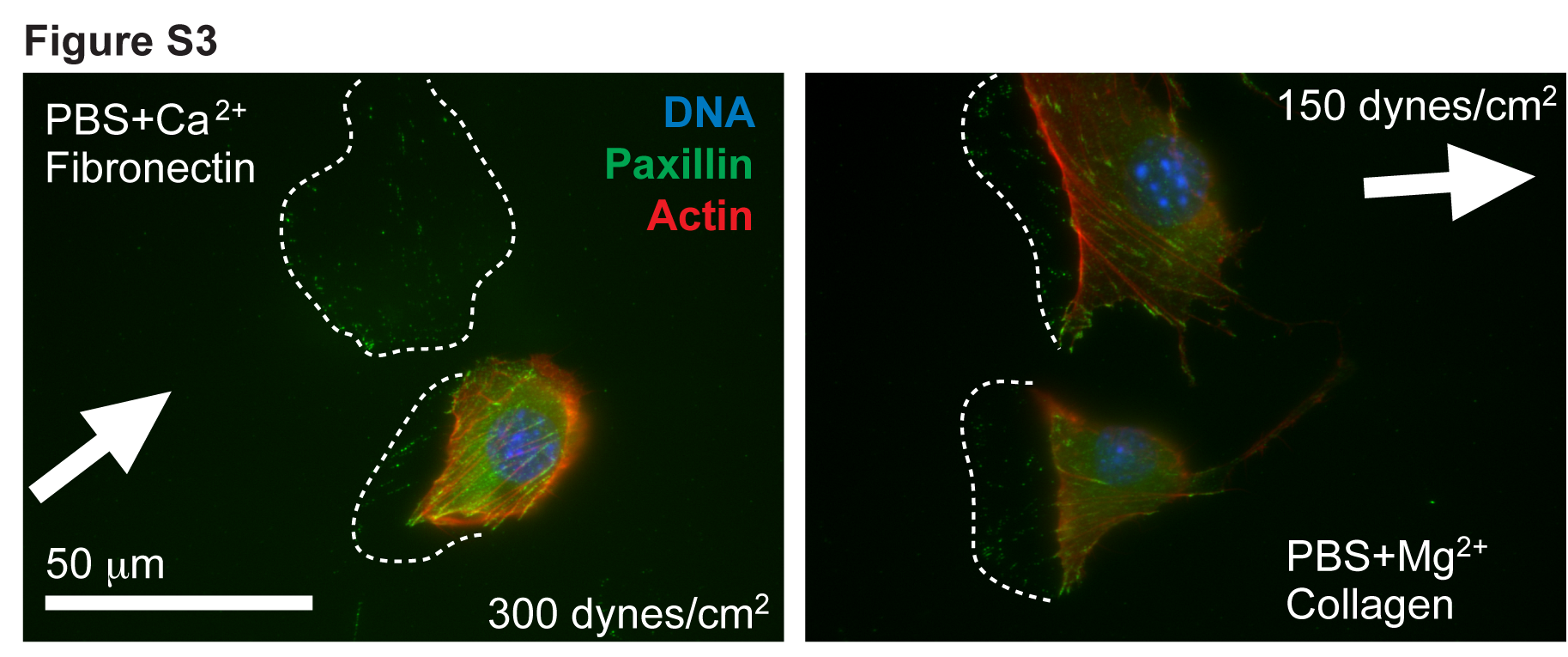

Supplement: Figure S3 — Shear-induced Cell Remodeling for Non-Aligning Conditions. 3T3 fibroblasts are shown under the indicated cation and ligand conditions. Shear direction in each image is indicated by a white arrow. Images show paxillin in green, the actin cytoskeleton in red, and the nucleus (DNA) in blue. The approximate pre-shear cell area is indicated by white dashed lines as determined from the focal adhesions that remained on the substrate. (TIF) [file pone.0102424.s003.tif]

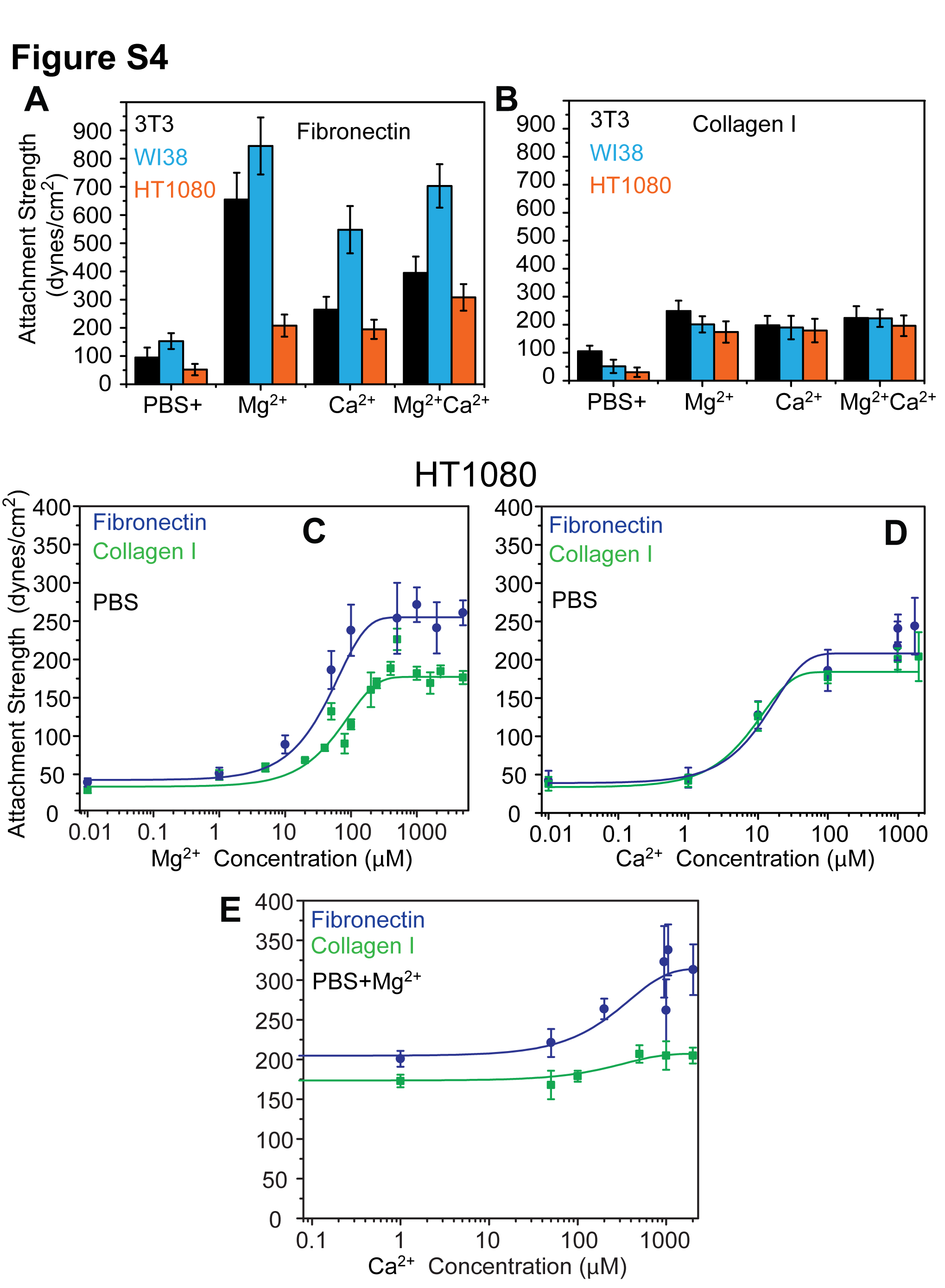

Supplement: Figure S4 — Quantification of Shear-induced Cell Remodeling for Non-Aligning Conditions. (A-B) Attachment strength of 3T3, WI38 and HT1080 cells under the indicated cation and ligand conditions. (C) Adhesion strength, T 50 (measured in dynes/cm2), for HT1080 cells on fibronectin- (blue) and type I collagen-coated substrates (green) in absence of calcium but in the presence of 0.01–1000 µM Mg2+. Data are fit by sigmoidal curves. (D) Adhesion strength, T 50 (measured in dynes/cm2), for HT1080 cells on fibronectin- (blue) and type I collagen-coated substrates (green) in the presence of 1–1000 µM Ca2+ without Mg2+ present. Data are fit by sigmoidal curves. (E) While keeping Mg2+ constant at 0.5 mM, adhesion strength was measured as a function of Ca2+ for both fibronectin- (blue) and type I collagen-coated substrates (green). (TIF) [file pone.0102424.s004.tif]

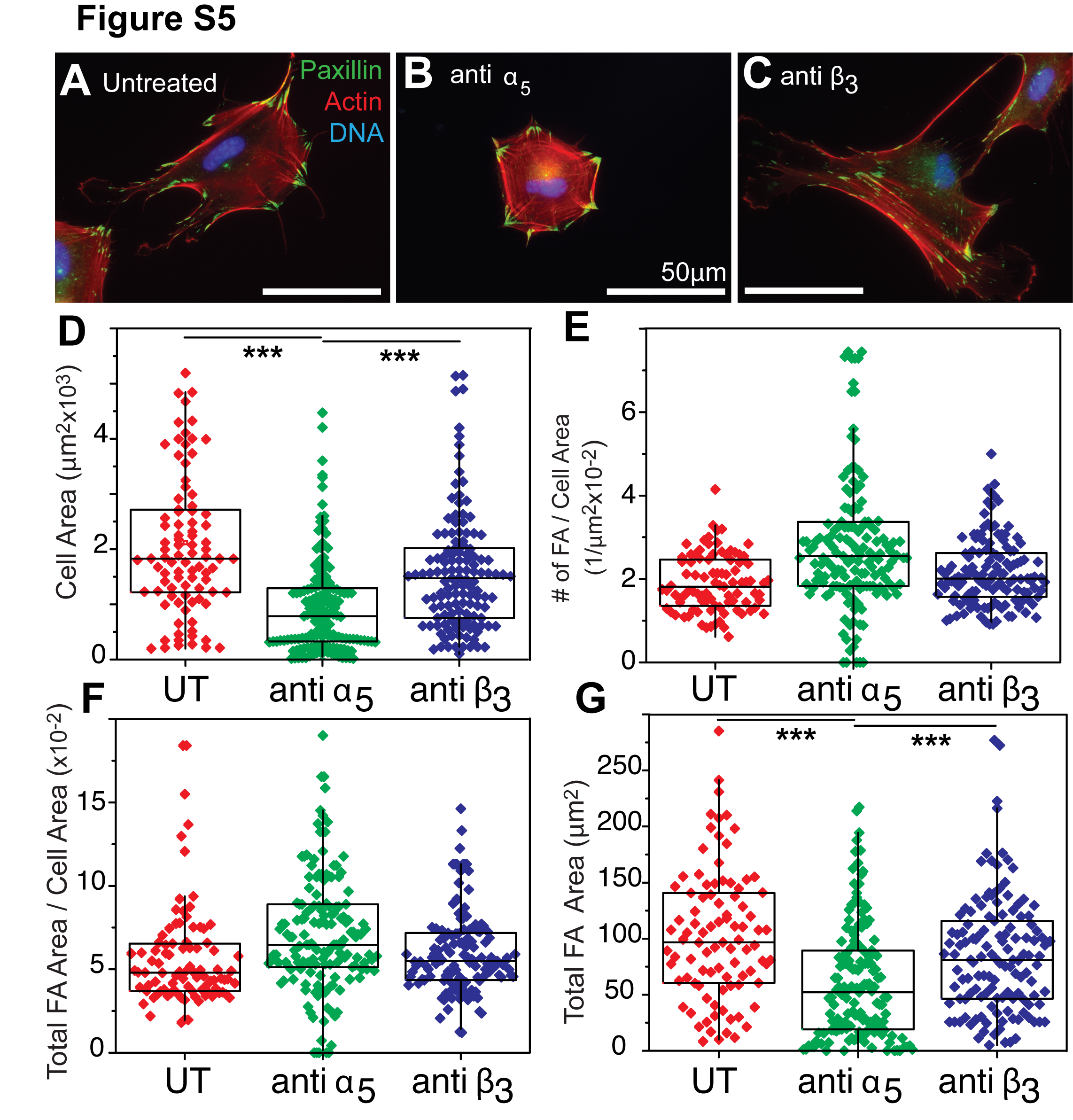

Supplement: Figure S5 — Blocking α5 but not αv Integrin Function without Shear in Magnesium-containing Media alters Attachment of WI38 Fibroblasts. (A-C) 60x fluorescence images of WI38 fibroblasts 2 hours post-seeding on fibronectin showing paxillin (green), actin (red) and DNA (blue). Inset images are shown from regions outlined in white. Cells were treated with the indicated conditions: (A) WT, (B) blocking α5 integrins, and (C) blocking β3 integrins. (D-G) Quantification of indicated morphological and FA parameters for the same conditions in panels A-C performed in triplicate. * p<0.05, *** p<0.001. 10x fluorescence images of WI38 fibroblasts, actin (red) and DNA (blue), after cyt D treatment (bottom) and without (top) as well as low (left) and high (right) application of shear. Direction of applied shear indicated by arrow. (TIF) [file pone.0102424.s005.tif]

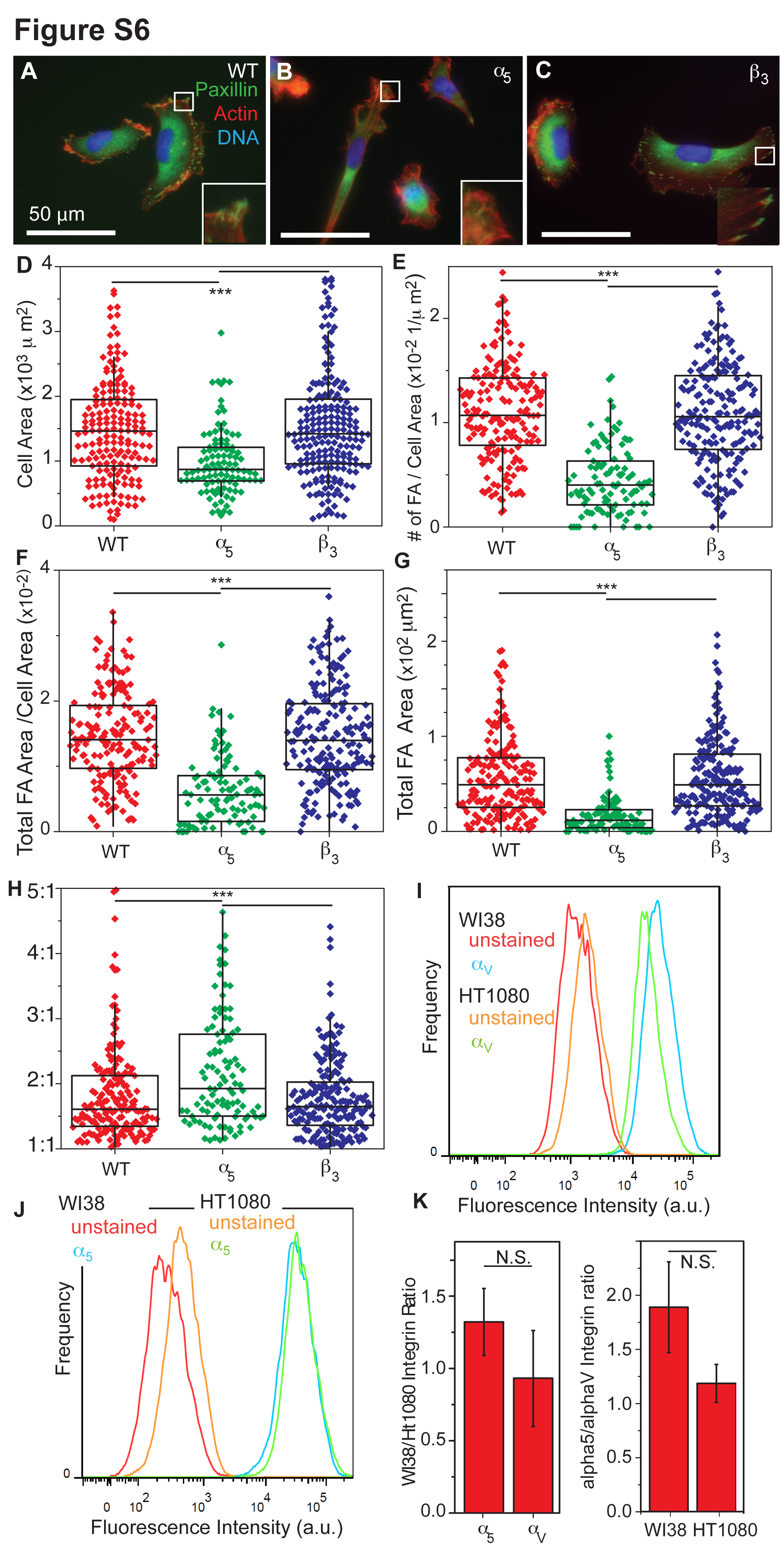

Supplement: Figure S6 — Blocking α5 but not αv Integrin Function without Shear in Magnesium-containing Media for HT1080 Fibrosarcoma Cells. (A-C) Fluorescence images of HT1080 fibrosarcoma cells 3 hours post-seeding showing paxillin (green), actin (red) and DNA (blue). Inset images are shown from regions outlined in white. Cells were treated with the indicated conditions: (A) WT, (B) blocking α5 integrins, and (C) blocking β3 integrins. (D-H) Quantification of indicated morphological and FA parameters for the same conditions in panels A-C. (I-J) Flow cytometry comparing α5 and αV integrin expression peaks for WI38 fibroblasts and HT1080 fibrosarcoma cells. (K) Shown are ratios of integrin subtypes within a single cell type (left) and for a single integrin subtype between cell types (right). *** p<0.001, N.S. = not significant. (TIF) [file pone.0102424.s006.tif]
