# Supplementary material for: Cation Type Specific Cell Remodeling Regulates Attachment Strength
Source: PLoS One. 2014 Jul 11;9(7):e102424. doi: 10.1371/journal.pone.0102424 (PMC4094514; doi:10.1371/journal.pone.0102424)
Supplement: Table S1 — Standard media formulations for each cell type used with Dulbecco's modified Eagle's medium (DMEM) are listed. Additional components and concentrations not specifically mentioned here are 4 mM L-glutamine, 1 mM sodium pyruvate, and 100 U/mL penicillin. The table specifically notes standard cation concentrations in commercially available solutions of DMEM and serum (column 3; [42]) and the range tested (column 4), with specific concentrations indicated in the text. (DOCX) [file pone.0102424.s007.docx]

**Cation Type Specific Cell Remodeling Regulates Attachment Strength**

Alexander Fuhrmann^1^, Julie Li^1^, Shu Chien^1-3^, and Adam J. Engler^1,3*^

Departments of ^1^Bioengineering and ^2^Medicine, and Institute of Engineering in Medicine; University of California, San Diego; La Jolla, CA 92093, USA

^3^Sanford Consortium for Regenerative Medicine; La Jolla, CA 92037, USA

*Correspondence: [aengler@ucsd.edu](mailto:aengler@ucsd.edu)

Phone: 858-246-0678

Fax: 858-534-5722

**MANUSCRIPT INFORMATION**

*Abstract Count*: 148 words

*Word Count*: 6,093 (References excluded)

*Figure Count*: 8

*Supplemental Figure Count*: 5

*Author contributions*: A.F. performed research, analyzed data, and contributed new analytical tools; J.L and S.C. modified the fluid flow chamber and provided technical assistance; A.F. and A.J.E. designed research and A.F., A.J.E and S.C wrote the paper.

*Keywords*: Adhesion, Spinning Disc, Shear, Fibroblast, Fibrosarcoma

*Running titles*: Cation-Dependent Detachment

**Supplemental Tables**

**Table S1:** Standard media formulations for each cell type used with Dulbecco’s modified Eagle’s medium (DMEM) are listed. Additional components and concentrations not specifically mentioned here are 4mM L-glutamine, 1mM sodium pyruvate, and 100 U/mL penicillin. The table specifically notes standard cation concentrations in commercially available solutions of DMEM and serum (column 3; [42]) and the range tested (column 4), with specific concentrations indicated in the text.

| **Cell Type** | **Glucose (g/L)** | **Serum (% v/v)** | **Cation (mM)** | **Cation Range (mM)** |
| --- | --- | --- | --- | --- |
| NIH 3T3 Fibroblasts (murine) | 4.5 | 10 (Fetal Calf Serum) | Ca+: 1.8 mM  Mg+: 1.0 mM | Ca+: 0–2 mM  Mg+: 0 –1 mM |
| WI38 2RA Fibroblasts (human) | 1.5 | 10 (Fetal Bovine Serum) | Ca+: 1.8 mM  Mg+: 1.0 mM | Ca+: 0–2 mM  Mg+: 0–1 mM |
| HT1080 Fibrosarcoma cells (human) | 4.5 | 10 (Fetal Bovine Serum) | Ca+: 1.8 mM  Mg+: 1.0 mM | Ca+: 0–2 mM  Mg+: 0–1 mM |
